# Progress in Medicine

**Published:** 1899-03-18

**Authors:** 


### Progress in Medicine.

### DISEASES OF RESPIRATORY SYSTEM.

**Pneumonia.**—Eyre and Washbourne[1] have by a series of experiments tested the virulence of certain varieties of pneumococci. The two extremes are the parasitic and the saprophytic cocci, the former being extremely virulent and the latter very feebly so. An intermediate variety was obtained from the sputum of a person in ordinary health. This was capable of growing at 20°C., and only reached standard virulence after passage through 53 rabbits, rapidly losing its virulence in cultivations. In describing varieties of pneumococcus it is essential that the power of acquiring and of retaining a high degree of virulence must be taken into account. With an increased knowledge of the bacteriology of the disease, the claim of lobar pneumonia to be regarded as a specific fever is being more firmly established, and its treatment by some form of anti-pneumococcic serum is looked upon with greater favour. Smith[2] reviews the progress of the serum-therapy of pneumonia up to the present time. Whilst he believes that the crisis coincides with cessation of activity of the pneumococcus, yet the production of an antitoxin also plays a part. The earliest successful attempts at rendering animals immune to the disease were obtained by injecting into them the pleuritic exudation, spleen substance, or sputum of animals suffering from pneumonia. Foa first immunised by means of attenuated cultures of the diplococcus, and Klemperer later demonstrated the curative effects of serum taken from immunised animals. Lara, Bozzolo, De Renzi, and others witness to the success of the serum treatment of the disease. Washbourne has used the serum of an immunised pony with encouraging results. The New York Health Board is making efforts to supply an anti-pneumococcic serum. As a prophylactic the serum treatment is not so promising, the period of conferred immunity being very short.

In an article entitled "Chloride Metabolism in Pneumonia,"[3] Hutchinson states: (1) That in croupous pneumonia the chlorides in the urine are diminished or entirely disappear; (2) that these chlorides are retained in the body; (3) following the crisis by some two or three days there is an excessive secretion of chlorides; (4) phosphates and sulphates are increased in the urine. Although this suppression of chlorides may take place in the course of other infective fevers, croupous pneumonia is the only pulmonary disease in which it is met with to any appreciable extent, and, therefore, an examination of the urine may prove of great value in distinguishing pneumonia from other conditions, such as empyema, pleurisy, &c. The degree of suppression is of no help in forming a prognosis. It is noteworthy that, although the sputa in pneumonia are rich in chlorides, the total amount secreted daily by this channel is small. The pulmonary exudation does not contain a large quantity of the chlorides, and the quantity in the blood is less than in health. The chlorides generally seem to be retained in all the organs and the fixed tissues, its presence there being probably necessitated by the increased metabolism associated with the fever.

Of the many complications of pneumonia, that described by Da Costa[4] is one of the rarest. He relates the details of three cases of phlegmasia alba dolens following acute lobar pneumonia, and has collected six other recorded cases. The internal saphenous or the femoral vein of the left leg is the most frequent seat of the lesion, and it is uncertain whether the co-existing phlebitis is cause or effect. The altered blood condition and languid circulation occurring at the end of the acute infectious malady is probably the predisposing cause. Prognosis is favourable, but one fatal case is recorded. A case of acute mania complicated by pneumonia is recorded by Rosenthal.[5] Post-mortem examination showed double lobar pneumonia. The exudation, which was of a peculiar mucoid character, gave rise on culture to pneumococci, pneumobacilli, and streptococci. There was, in addition, double fibrinous pleurisy, pachymeningitis, and acute purulent leptomeningitis, the organism found in the meningeal exudation being streptococcus. Rosenthal believes that the original lesion was bronchitis, and a septic infection by streptococcus. This infection spread to the cortex, exciting the maniacal condition, and the pneumonia was a secondary invasion. A very similar case is that reported by Aufrecht.[6] Together with pneumonia, there was pleural effusion, followed a week later by acute cerebral symptoms of one day's duration. Subsequently the patient developed an empyema, and after this was opened made a good recovery and was discharged. Eight days later there was a return of the pyrexia, with marked meningeal symptoms. Coma preceded death. The post-mortem examination showed that there had been rupture of an abscess in the left corpus striatum, and the consequent purulent meningitis had caused death.

**Idiopathic Pulmonary Congestion.**[7]—This disorder, sometimes known as Woillez's disease, is an acute affection following exposure to cold or resulting from injuries to the chest wall. The earliest symptoms—slight shivering and pain in the side—are noted from four to twelve hours after exposure to the exciting cause. The rigors may last for three or four hours, but are never severe and prolonged as in pneumonia. The pulse is rapid, respiration frequent, and temperature high. Cough and expectoration are slight. Physical examination may show the side of the chest affected to be increased in size, with impairment of movement. Resonance on percussion is impaired, and with prolonged expiration there may be heard crepitant râles. A distinctive feature of the disease is a form of echo immediately following voice sounds; this is known as Serrand's sign. The duration of the disease is from four to five days, and relapses are common. Occasionally pneumococci are found in the sputa, but more commonly staphylococci. The disease differs from pneumonia in that the lung tissue, although congested, is not solidified. Pneumococci may, however, be found in such patches or in the organs after death.

**Acute Pulmonary Œdema.**—A case of acute pulmonary œdema is described by Whitney.[8] The patient was a woman 25 years old, with disease of both mitral and aortic valves, with dilation of ventricles and failure of compensation. The signs of the œdema were of sudden onset—cyanosis, dyspnœa, and a flow of white frothy sputum from the mouth. On auscultation coarse and fine moist râles were heard over both lungs. Cyanosis and air-hunger increased, and there was an enormous discharge of blood-stained sputum. The removal of 12 ounces of blood by venesection gave immediate relief, and the patient made a speedy recovery. Venesection is not indicated in cases of general anasarca, with cardiac insufficiency and engorgement of the lungs, but when the pulmonary œdema is sudden and excessive—an accident, as it were, resulting from some unusual strain upon the heart, and upon a heart which is capable of some recuperative effort.

**Empyema.**—Hutton[9] advocates more active measures to ensure the rapid closure of empyema cavities. At the present time cases run for months or years, and complete restoration of function is exceptional. Whilst repeated aspirations may suffice in the case of children, incision and drainage is necessary in adults, though it is often advisable to aspirate first. The best spot for the incision is over the sixth rib in the midaxillary line, for this is the part to which the lung last expands; and as the patient lies on his back the tube will not be pressed upon. The two chief indications are to provide an exit for the pus and to aid the expansion of the lung. These, the writer believes are best brought about by the use of a duckbill valve. Details of the arrangement are given, and it is claimed that it is devoid of risk, simple, inexpensive, and comfortable, and has a marked effect in hastening recovery. It is not suitable when an opening into the lung exists. Syphon drainage also hastens lung expansion. Irrigation of empyemata[10] is sometimes followed by unpleasant and dangerous symptoms. In one case, after using a wine-glassful of peroxide of hydrogen, there was loss of consciousness, transient paralysis of the right side, and difficulty in breathing. In another, after the use of a solution of iodine, the patient was seized with malaise, numbness of the right side of the body, and loss of speech. Sudden cessation of heart beat and of respiration, convulsions, collapse, hemiplegia, and sudden death are other complications reported. Janeway regards the symptoms as due to emboli of air or gas, but it is equally possible that the emboli may be solid bodies so small as to have been overlooked in those cases in which a necropsy has been held. Irrigation of the pleura is, if possible, to be avoided.

[1] Lancet, Jan. 7, 1899. [2] Amer. Jour. Amer. Sci., Oct., 1898. [3] Jour. Path. and Bact., Dec., 1898. [4] Philadelphia Med. Sur., Sept. 10, 1898. [5] Munchener Med. Woch, Oct., 1898. [6] Deutz. Archiv. fur Klin. Med. Bd. ix., heft. 5. [7] Rev. de Méd., Oct., 1898, Carrière. [8] Med. News, Dec. 31, 1898. [9] Brit. Med. Jour., Oct. 29, 1898. [10] Ibid, Nov. 26, 1898.

